# Urban Physiology: City Ants Possess High Heat Tolerance

**DOI:** 10.1371/journal.pone.0000258

**Published:** 2007-02-28

**Authors:** Michael J. Angilletta, Robbie S. Wilson, Amanda C. Niehaus, Michael W. Sears, Carlos A. Navas, Pedro L. Ribeiro

**Affiliations:** 1 Department of Ecology and Organismal Biology, Indiana State University, Terre Haute, Indiana, United States of America; 2 School of Integrative Biology, University of Queensland, St Lucia, Queensland, Australia; 3 Department of Physiology, Institute of Biosciences, University of São Paulo, São Paulo, Brazil; 4 Department of Zoology and Center for Ecology, Southern Illinois University, Carbondale, Illinois, United States of America; University of Stellenbosch, South Africa

## Abstract

Urbanization has caused regional increases in temperature that exceed those measured on a global scale, leading to urban heat islands as much as 12°C hotter than their surroundings. Optimality models predict ectotherms in urban areas should tolerate heat better and cold worse than ectotherms in rural areas. We tested these predications by measuring heat and cold tolerances of leaf-cutter ants from South America's largest city (São Paulo, Brazil). Specifically, we compared thermal tolerances of ants from inside and outside of the city. Knock-down resistance and chill-coma recovery were used as indicators of heat and cold tolerances, respectively. Ants from within the city took 20% longer to lose mobility at 42°C than ants from outside the city. Interestingly, greater heat tolerance came at no obvious expense of cold tolerance; hence, our observations only partially support current theory. Our results indicate that thermal tolerances of some organisms can respond to rapid changes in climate. Predictive models should account for acclimatory and evolutionary responses during climate change.

## Introduction

Major cities are hotter than their surroundings for many reasons, including the greater radiation from surfaces, the greater emission of heat, the thermal mass of buildings, the reduced evapotranspiration from soil, and the unusual pattern of convection [1]. Urban heat islands—increased temperatures within urban areas—scale logarithmically with the population of a city [2]. In the world’s largest cities, the difference between urban and rural temperatures reaches as much as 12°C. Urban warming likely has widespread biological consequences; after all, environmental temperature has been linked to everything from temporal patterns of growth, survival and reproduction [3]–[5] to spatial patterns of body size, population density, and species diversity [6]–[8]. Urban heat islands should not only concern ecologists who wish to manage urban populations, but they should also interest physiologists who seek to test theories of thermal adaptation.

Optimality models predict acclimatory or evolutionary responses, such that ectotherms in warm environments should tolerate high temperatures better and low temperatures worse than ectotherms in cool environments [9]–[11]. Researchers usually test these predictions by comparing indices of acute or chronic thermal tolerance, such as the duration of survival during exposure to an extremely high temperature (knock-down resistance) or the duration of recovery after exposure to an extremely low temperature (chill-coma recovery). For several species, researchers have shown genotypes from low latitudes or altitudes tolerate heat better but cold poorer than do genotypes from high latitudes or altitudes [12]–[14]. Therefore, we suspected similar differences in thermal tolerance would be associated with the more localized clines in temperature caused by urbanization. Specifically, ectotherms from warm, urban environments should tolerate heat better and cold worse than ectotherms from cool, rural environments [15].

**Figure 1:**
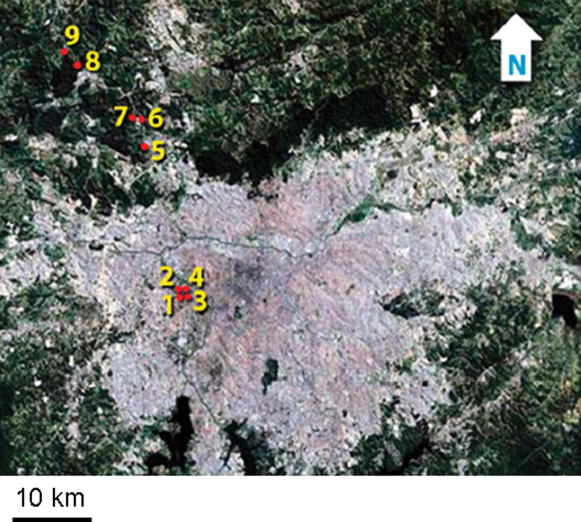
A satellite image of São Paulo showing the location of the colonies that we sampled for our study (image available from the United States Geological Survey, Sioux Falls, SD). Colonies 1–4 and 5–9 experienced urban and rural environments, respectively.

**Figure 2:**
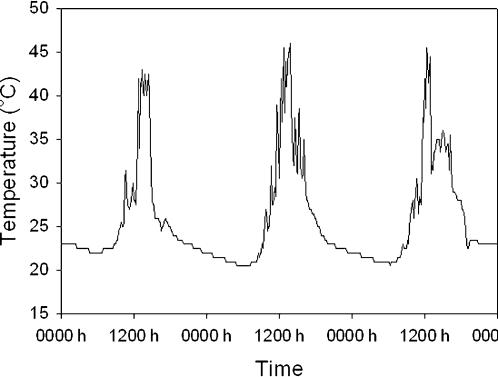
Trail temperatures exceed the critical thermal maximum for leaf-cutter ants for several hours per day. These data were recorded by an iButton Thermochron (Dallas Semiconductors) that was placed in full sun on the surface of an active trail. Data were collected during the week preceding our study (19–22 March 2006); this same trail should be even hotter during the middle of summer.

To test our predictions, we compared the thermal tolerances of leaf-cutter ants (*Atta sexdens*) from colonies inside and outside of São Paulo, Brazil (Fig. 1). With a population exceeding ten million people, São Paulo fuels one of the most intense urban heat islands in the world [16], with distinct peaks occurring during the night in winter and during the day in summer [17]. Surface temperatures of the urban heat island should determine the body temperatures of small, terrestrial organisms, such as leaf-cutter ants [18]. Although leaf-cutter ants emerge primarily at night, some ants also forage during the day when surface temperatures can exceed 45°C (Fig. 2). Tolerance of extreme heat would enable ants to evacuate a trail effectively as temperatures rise during mid-day.

## Methods

### Sampling of colonies

We sampled colonies of leaf-cutter ants (*Atta sexdens rubropilosa*) inside and outside of São Paulo. Our samples included four urban colonies near the University of São Paulo and five rural colonies distributed along a north-westerly transect (Fig. 1). We only collected ants that were walking along trails, carrying leaves or pieces of leaves. To minimize environmental and procedural effects, we collected all ants and measured their thermal tolerances on the same day (30 March 2006). We collected ants between 1600 and 2200 h and began measures of thermotolerance by 2300 h. Logistically, we were unable to collect ants from urban and rural colonies at the same time; therefore, urban ants were collected after rural ants to minimize the chance that high temperatures during collection would cause urban ants to tolerate heat better than rural ants. Between the time of collection and the time of the experiment, we held ants in large plastic jars containing a piece of saturated cotton as a source of water.

### Heat tolerance

We defined heat tolerance as the time required for ants to lose mobility at a stressful temperature, usually referred to as knock-down resistance [19]. This common measure of heat tolerance seems to correlate well with other measures of heat tolerance when comparing species [20]. During a preliminary study, we determined ants from urban colonies were immediately immobilized at 44°C ( = critical thermal maximum). We also noted that ant trails in the city approached these temperatures during mid-day (Fig. 2). Therefore, we assessed knock-down resistance at 42°C, a temperature slightly below the critical thermal maximum and within the range of temperatures experienced in nature.

To compare knock-down resistances of urban and rural ants, we used ants from four urban colonies (n = 24–30 per colony) and five rural colonies (n = 30 per colony). Ants from each colony were divided into groups of six; each group was placed in a Petri dish (150×10 mm) with a small piece of water-saturated cotton. The water was provided to ensure ants succumbed to heat instead of dehydration. During the trials, some ants were observed drinking from the cotton and sharing fluids with neighboring ants. Furthermore, heat tolerances of urban and rural ants were unrelated to body mass in either urban or rural colonies, suggesting variations in water stores and dehydration rates were unimportant; this conclusion was based on ANCOVA, in which colony was used as a random factor to control for pseudoreplication (urban ants: *β* = 4.6±4.0, *F*_1,5.9_ = 1.66, *P* = 0.10; rural ants: *β* = 0.41±1.19, *F*_1,8.2_ = 1.15, *P* = 0.31).

Knock-down resistance was measured in a walk-in environmental chamber that maintained a constant temperature during the experiment. All Petri dishes were brought into the room at the same time and were placed on a table for observation. Surface temperature of the table (42±1°C) was monitored with data loggers (i-Button Thermochron, Dallas Semiconductors) and an infrared thermal gun (Raynger ST30, Raytek). To minimize artifacts caused by thermal gradients, we created five zones along the length of the table; one Petri dish of ants from each colony was placed in each zone. Urban and rural ants within each zone were stratified to further reduce the chance of spatial artifacts.

During the experiment, at least four people scanned the dishes to identify the time that each ant lost mobility. Although ants clearly became disoriented before losing mobility, we found the loss of mobility to be a more objective criterion for scoring heat tolerance. Because ants can assume a curled position during heat stress, we tapped the dishes periodically to assess whether ants were truly immobilized or were merely exhibiting signs of stress. Tapping of dishes was performed in a systematic manner such that all ants were disturbed similarly.

We compared the knock-down resistances of urban and rural ants with a Cox proportional hazards model, from the survival library of the R Statistical Package (R Development Core Team 2006). Because knock-down resistances of ants within colonies and within Petri dishes were likely correlated, we used a robust sandwich estimate of the variance, which accounted for correlated responses of individuals within colonies and dishes [21], [22]. This procedure enabled us to generalize our findings without pseudoreplication.

### Cold tolerance

We defined cold tolerance as the time required to recover from exposure to 0°C, usually referred to as chill-coma recovery. To compare chill-coma recoveries of urban and rural ants, we used ants from four urban colonies (n = 19–31 per colony) and four rural colonies (n = 20–30 per colony). Ants were sorted into Petri dishes (150×10 mm), which were entombed in ice for a period of 20 min. After this exposure, dishes were removed from ice and ants were transferred to sheets of paper at room temperature (25±1°C). Using forceps, we positioned each ant on its back in the center of a printed circle (diameter = 32 mm). We recorded the times elapsed between the removal of dishes from ice and the recovery of each ant. Recovery was scored when an ant assumed an upright position and broke the plane of the circle; this simple, objective measure of recovery reflected the onset of motor coordination because ants generally began exploring their environment immediately after assuming an upright position. As each ant left its circle, we covered it with a small plastic cup to prevent the ant from interfering with other ants on the same sheet.

Our experimental design controlled for many sources of variation. Because ants had to be assayed in successive trials, each trial involved ants from one urban and one rural colony. To ensure accurate detection or recovery, no more than thirty ants from each colony were assayed at once, and at least three people watched the ants at all times. Petri dishes containing urban and rural ants were chilled together, and the position of urban and rural dishes was rotated between trials. To control for thermal heterogeneity within the room, we switched the positions of the papers for urban and rural ants between each trial. To minimize delays during data collection, we used event-recording software (ETHOM) to record the time of each observation [23]. We compared the times required to recover from chill coma using the same proportional hazards model that we used to compare knock-down resistances.

## Results

**Figure 3:**
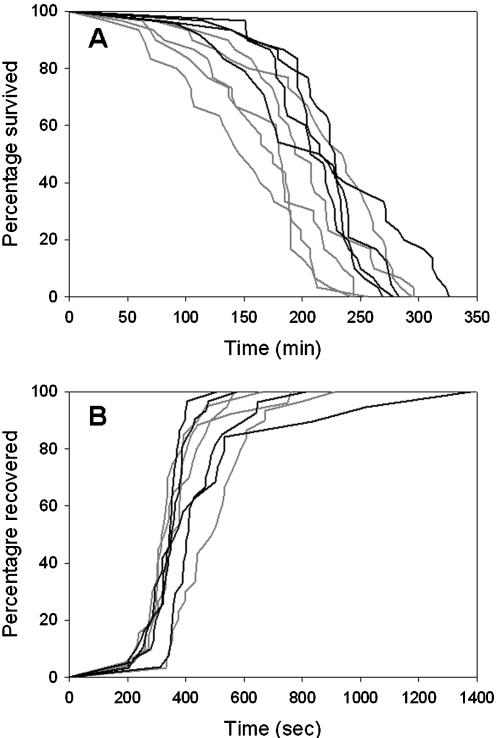
**A)** Ants from urban colonies (black lines) tolerated extreme heat (42°C) for longer than did ants from rural colonies (grey lines). Each line represents the survivorship curve of ants from a single colony. B) Ants from urban and rural colonies recovered from chill coma within a similar period of time. Each line represents the recovery curve of ants from a single colony.

When exposed to the stressful temperature of 42°C, ants from colonies within São Paulo survived 20% longer than ants from colonies surrounding São Paulo (mean±standard error  = 216±4.8 and 179±4.2 min for urban and rural ants, respectively). A Cox proportional hazards model indicated that rates of mortality differed significantly between urban and rural colonies (*β* = −0.535±0.237; *P* = 0.02; Fig. 3.A). This greater heat tolerance came at no obvious expense of cold tolerance; mean times for urban and rural ants to recover from chill coma were nearly identical (6.6±0.2 and 6.7±0.2 min for urban and rural ants, respectively). A Cox proportional hazards model indicated no significant difference between the rates at which urban and rural ants recovered from cold exposure (*β* = −0.124±0.288; *P* = 0.67; Fig. 3B). Hence, our observations only partially support current theory.

## Discussion

The difference in heat tolerance between urban and rural ants resembles differences documented within species that span latitudinal and altitudinal clines [reviewed by 24]. In *Drosophila melanogaster*, flies from northern Australia resisted heat longer but recovered from cold slower than flies from southern Australia [14]. In other species, either heat or cold tolerance varies geographically. For example, Sorensen and colleagues [25] observed an altitudinal cline in heat tolerance among populations of *Drosophila buzzatii*, without observing a cline in cold tolerance. This macrogeographic pattern observed among flies parallels the microgeographic pattern we observed among ants.

Currently, we do not know whether the difference between the heat tolerances of urban and rural ants has a genetic basis or simply results from plastic responses to environmental conditions. Studies of *Drosophila* have generally focused on offspring raised in a common environment to reduce environmental sources of variation in thermal tolerance. Unfortunately, we could not perform such studies with ants. Therefore, some or all of the difference in heat tolerance could have resulted from acclimatization of urban ants (i.e., heat hardening). Nevertheless, plasticity in heat tolerance could also be an adaptation to the mean and variance of environmental temperature [10], [11]. Whether genetic effects, environmental effects, or both caused the greater heat tolerance of urban ants, our finding suggests urbanization has influenced the phenotypes of ants in São Paulo.

The ecological significance of variations in knock-down resistance and chill-coma recovery remains controversial [19]. We believe these indices of thermal tolerance correlate with the thermal limits to survivorship under chronic exposure. Variation in these indices could also reflect variation in the shape of thermal performance curves, especially if performance at extreme temperature trades off with performance at intermediate temperatures [26]. Heat tolerance should directly benefit ants during activity because trails attain stressful temperatures for several hours per day (see Fig. 2). Indeed, many ants became disoriented and assumed defensive postures well before losing mobility, suggesting high heat tolerance would enable ants to evacuate a trail effectively as temperatures rise during mid-day. Observations of urban and rural ants in outdoor enclosures would help to define the ecological significance of the thermal tolerances that we observed.

Urban environments serve as excellent natural experiments for quantifying the impact of climate change on organisms. Although urbanization affects more than just the temperature of the environment, large cities arguably provide a timely model for understanding ecological and evolutionary responses to regional and global climate change. We expect researchers will find variations in thermal tolerance within other species that encounter urban heat islands. By studying such phenomena, scientists can better gauge the potential for acclimatization or adaptation during global climate change. Ultimately, studies of urban physiology could alter our current perspective, especially since most documented responses to climate change involve shifts in behavior and phenology rather than shifts in physiological tolerance [27].
